# Saint Louis and the Exposition of 1904

**Published:** 1903-07

**Authors:** Nelson W. Wilson

**Affiliations:** Buffalo, N. Y., Genitourinary Surgeon, Buffalo Emergency Hospital. 49 Niagara Street


### SPECIAL ARTICLE.

### Saint Louis and the Exposition of 1904.

By NELSON W. WILSON, M. D., Buffalo, N. Y.,
Genitourinary Surgeon, Buffalo Emergency Hospital.

IF ONE recalls pre-Pan-American days in Buffalo, when all eyes and thoughts were turned toward the embryonic City of Light, he will have a very fair idea of the present condition of St. Louis, where they are getting ready for an exposition which, while larger in scope and more lavish in the expenditure of ready money, will not be more beautiful than our own Rainbow City, nor more excellently managed. The West does things on a large scale, and the exposition will not be an exception in any sense of the word. There are acres upon acres of land, lawns, plains, hills and wooded slopes which will be transformed into perfect bowers of beauty and loveliness when the artificers of a modern age have completed their handiwork. There will be places which will remind one of the Pan; and effects will bring back memories of delightful nights under the stars of electricity, for Henry Rustin is at work in the Mound City and is preparing wonders on wonders for the display of the marvels of electricity. And the midway, too, will be there, and the camels and the Turks and the Cingalese, and the rest of the naturally malodorous and unsanitary Oriental outfit, only at St. Louis there will be less trouble than there was at Buffalo, for the concession has been let to a gentleman with modern ideas and who promises to make his watchword "Cleanliness."

It was partly business and partly pleasure which took me to St. Louis recently. Naturally, I noticed the country, the farms and the types of people and the general breeziness and the lack of restraint, and the increasing freedom as the Wabash railroad carried me a step or two nearer the land of the setting sun. The fact that I traveled like a gentleman and paid my fare makes reference to the railroad a compliment, and not an obligation; and of a truth the management deserves a word of praise. Not because the trains are more luxurious than those of other roads, but because they are on the whole more sanitary, interested me more than anything else in regard to them. I suppose that because I was going from one exposition city to another, memories of Pan-American days rushed on me, and I longed to see the kitchen of that train and the food boxes and cooking apparatus; that, I presume, is one of the legacies of the Pan; and, all unannounced, the dining car conductor took me forward and showed me a group of colored cooks busy at the fires and pointed out the highest type of a cleanly and sanitary kitchen; where the cooking was done in a human manner and nothing was pawed over; and during the few moments I was in the place of appetizing dishes I saw the cooks wash and rinse their hands in clean water, not once, but many times. Thereafter I ate in the dining car with an easy mind.

Of course, everyone got a look at "the bridge" as the train went into St. Louis. There were a lot of expectant concessionaires on the train, a few "show men," who were on a still hunt for jobs; some others who were going on; and one Buffalo man who had "rheumatism" and was en route to Hot Springs to get "boiled out," and who damned everything with exposition attached to it. He wouldn't stop over and look at the grounds, not he. He had had an exposition in his own town and he was sick of them; they were no good, and he wanted none of them in his. And no wonder, for he was one of the annointed of the croaker's club, which flourished in Buffalo during the entire exposition period, and this same man used to go into the grounds on a laborer's day pass, although he had money sufficient to live at his ease without work and sufficient besides to acquire "rheumatism." I imagine he got properly "boiled out," for recently I have seen him at home with a clear skin.

The little bit of business which took me to St. Louis was responsible for the spoiling of a great deal of pleasure; for hospitality was everywhere marked. When you go to St. Louis, be prepared to give yourself into the hands of your friends or displease them. St. Louisans take you bodily and make you one of them; you are at home. In some other cities you may be made much of if you're a big man; but if you have an introduction into St. Louis you are made much of even if you are only a little fellow in the profession. The doctors in that town take you by the hand and look you in the eye and say, "I am glad to know you;" and you feel that they are and that they haven't got a string to their welcome. Everywhere there is that delightful tinge of Southern accent, which means hospitality. There is a cheerful lack of narrowness either in business or society. The first impression is that the people are happy and prosperous, and they are, except when they get tangled up with one of the trolley cars, and then they get materially the worst of it. The trolleys in St. Louis make good time and incidentally help the undertaker. There were two persons killed during the three days I was in St. Louis, and only the company knows how many were injured. I was told by a doctor who is in position to know, that there were over 100 deaths and nearly 5,000 injured persons to the credit of the hungry trolley cars in St. Louis during the preceding year. Buffalonians do more or less kicking over the trolleys here, but we have no such bloody record as that, and the St. Louis company might with advantage to the people of the Mound City take lessons in management from Mr. Caryl Ely and his assistants in Buffalo.

I met Dr. L. H. Laidley, Medical Director of the St. Louis Fair, at the Pan-American, which he visited to look over the hospital and medical department, and the renewing of the acquaintance in St. Louis was most delightful. Naturally, Dr. Laidley is most deeply interested in preparing for the great crowds which are expected. He has a monumental task on his hands and he realises it. The conditions in St. Louis are widely different from those in Buffalo; everything will be on a grander scale; there is more money available, and to the credit of President Francis be it said that he is willing to spend lavishly where the hospital and medical department is concerned. The hospital will be located near the entrance and will be larger than the Pan-American institution; it will need to be, too, for St. Louis is a hot town in summer and the heat will keep the house and ambulance staffs busy. Then, too, I think I may be pardoned my excessive pride, when I say that Buffalo is a far more sanitary city than St. Louis. Dr. Wende, as health commissioner, did much to assist Dr. Park in maintaining sanitary excellence at the Buffalo exposition.

St. Louis is, moreover, notoriously corrupt in a civic sense, and being throttled in the grasp of grafting politicians it is not at all certain that money appropriated by the city authorities for sanitary purposes, will be used honestly if handled by politicians. In that way Dr. Laidley is handicapped. He is a wide-awake, energetic man, full of sturdiness and high ideals; he is pushing and forceful, and plows ahead to a given point in his work, no matter how great the obstacles or how apparently insurmountable. He is doing everything possible to preserve the health of the beautiful city-to-be, and should the great misfortune of widespread disease make its appearance during the life of the exposition, he will be the man to meet and handle the emergency. Nothing but the handiwork of fate and a rotten political administration can prevent success, for Dr. Laidley and the officers of the fair are doing all in their power to make things clean and keep them so. Dr. Martin E. Sheets is the sanitary officer during the construction period, and Dr. Joseph G. Moore is in charge of the emergency hospital on the grounds. Naturally everything is at odds and ends now, yet in spite of existing difficulties the work done at the hospital is excellent. Another member of the medical staff at the present time is Dr. L. P. Walbridge, who is visiting physician.

Just now serious efforts are being made by the medical profession to bring about a gathering of the brightest minds in medicine of the world during the exposition, as a World's Congress. The preliminary work is in charge of a committee on an International Medical Congress, and I was honored by an invitation to meet the committee at the home of one of its members, Dr. W. E. Fischel, the chairman. The committee has what might be called a social session before taking up the real work, at which they lay aside all the cares of the day. Dr. Fischel is much like Dr. Stockton in manner; pleasant, easy and self-contained, always inspiring confidence by the very ease of his bearing. Dr. Laidley is the serious side of the committee, although he has a fund of humor and is a well-spring of wit. Another member is Dr. Herman Tuholske, rather rotund, full of quaint sayings and oddities of speech; a laugh which makes him your friend; a man whose work in surgery has made him famous and whose rise in his profession reads like a romance; a close student and a hard worker. Dr. Frank J. Lutz was also present at the meeting. He is the surgeon of the gathering, a bright energetic man, whose every movement indicates the cool, alert, self-confident surgeon, that is, provided one knows he is a surgeon; otherwise one might say at first glance that he was a business man who controlled vast enterprises. These are the men who will bring about the congress if it is to be, and there is a fair prospect of success. It is designed to be one of the most remarkable gatherings in medical history, and when success crowns their efforts, as it assuredly must, New York State will be honored by able representation among the appointments.

At the St. Louis Club, with Dr. Laidley, I met the controlling spirits of the big fair, chief of whom is ex-Governor Francis, a man who does not impress one from casual observation as being possessed of such marvelous capacity for work as he has shown; nor as a man with more than the usual amount of executive ability. He sits low in a big, easy chair, and listens at times with a far-away look, apparently not paying the slightest attention to what is being said. He might be a star-gazer for all one could guess from looking at him; yet not a word escapes, and he absorbs every important detail of conversation; and when it is time for his decision he acts quickly and finally. There is no see-sawing with the president of the St. Louis Exposition. His grasp of affairs is marvelous, and he knows all the details of management of the various big expositions, with facts and figures at his tongue's tip; and while his aides carry slips of reminders of the business details of the St. Louis show, Mr. Francis has all his material stored away in the recesses of his mind ready for use at a moment's notice. It isn't hard when once you know the man and see him at work to understand why he is the absolute head, the dictator, of the movement. He sees everyone; no matter in connection with the work of the exposition is too small or too trivial for his consideration.

I imagine he has every salient point of every report in connection with the Pan indelibly fixed in his mind and ready for instant use. They were discussing the application of a concessionaire at Buffalo who was making efforts to get into St. Louis. His name was mentioned. Gov. Francis was looking nowhere in particular and giving little apparent attention to the discussion. When the concessionaire's matter had been threshed out he slowly, almost languidly, raised his eyes and said: "I don't think we had better consider that matter; Dr. Park's report at Buffalo did not speak very well of his concession if I remember correctly." The incident is mentioned merely to illustrate the type of mind which is controlling the workings of the fair. They are taking advantage of Buffalo's experience largely. Mr. Buchanan was there as the president's advisor, and the medical department was constantly referred to. Truly Governor Francis is a study and one marvels at the capacity of the man for work and his grasp of detail, when one considers that no important step is taken in the numerous departments in the beautifully classic Washington University buildings on the hill, which are being utilised as administration headquarters, without "the Governor" passes on it.

A fleeting visit to a city is unsatisfactory so far as hospital observation is concerned, yet during the brief time I spent in St. Louis I accompanied Dr. J. P. Bryson to the Mullanphy Hospital, under the management of the Sisters of Charity; a hospital which was the gift of the man for whom it was named; a man who came to St. Louis a poor and friendless Irish immigrant and worked with roustabouts on the levees. When he died he left an immense fortune, part of which went for the establishment and maintenance of the hospital, a splendid institution equipped with every convenience. Dr. Bryson is a typical Southerner with all the term implies, the gentility, the courtesy and the charming hospitality which characterises his people. Dr. Bryson's name is intimately connected with the advances in the surgery of the prostate; he has done an immense number of prostatectomies, and his success is second to none. He is a prolific writer on the subject and his descriptions of his own method are classics in the literature of genitourinary surgery. Dr. Bryson is professor of genitourinary diseases in Washington University medical department.

At the Marion-Sims-Beaumont medical college, Dr. Bransford Lewis is professor of genitourinary surgery. He is a young man, but he has done an immense amount of original work. Probably his most valuable contribution is his invention of the instrument for ureteral catheterisation, which is generally termed a ureter-cystoscope. It was my good fortune to see Dr. Lewis use the instrument in collecting samples of urine from each kidney, and the ease with which the operation was performed was amazing. There is absolutely no possibility of mixture of the urines, and at the same time a complete cystoscopic examination may be made.

Truly, St. Louis is a delightful city, looking at it professionally. From an expositional viewpoint it will be a wonder city; politically it is stricken with a grievous malady, although a brave and honest man, Attorney Foulke, is cutting into the rotten tissues with fearless hand; and it is a matter of professional pride that every physician in St. Louis, irrespective of school, college or society, is giving his aid to purge the town of its corruption. Dr. Laidley began a fight some time ago for a better water supply, and there are at last grounds for hope that with the assistance of his brother practitioners, and the decent element of the city, success will come and the health and lives of St. Louisians will not be subservient to the grafter's greed.

49 Niagara Street.

Note.—Since the above was written, Dr. Bryson has died; and in his demise the medical profession of St. Louis has lost one of its most prominent members, and the profession in general one of its most enthusiastic workers in the domain of special surgery. Reference to Dr. Bryson's death is made elsewhere in this issue.—Editor.

